# Supplementary material for: High-Performance, Easy-to-Fabricate, Nanocomposite Heater for Life Sciences and Biomedical Applications
Source: Polymers (Basel). 2024 Apr 20;16(8):1164. doi: 10.3390/polym16081164 (PMC11055136; doi:10.3390/polym16081164)
Supplement: Supplementary file 1 [file polymers-16-01164-s001.zip › polymers-2807005-supplementary.pdf]

polymers

Article

High-Performance, Easy-to-Fabricate, Nanocomposite Heater for Life Sciences and Biomedical Applications

Yudan Whulanza [1,2,*], Husein Ammar [1], Deni Haryadi [1,3], Azizah Intan Pangesty [2,4], Widoretno Widoretno [5], Didik Tulus Subekti [5] and Jérôme Charmet [6,7,*]

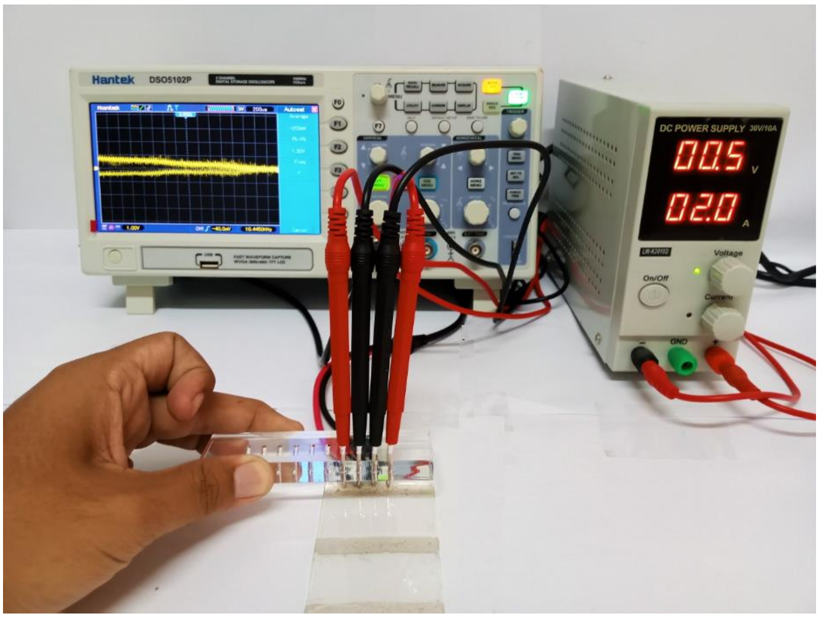

Figure S1. Four-point probe measurement set-up to measure the resistivity of the electrodes. The four-point probe method (also called the Kelvin method) consists of four equally spaced, co-linear electrical probes. Current is applied between the two outer electrodes and the voltage is measured between the two middle ones. This configuration eliminates contact and wire resistance.

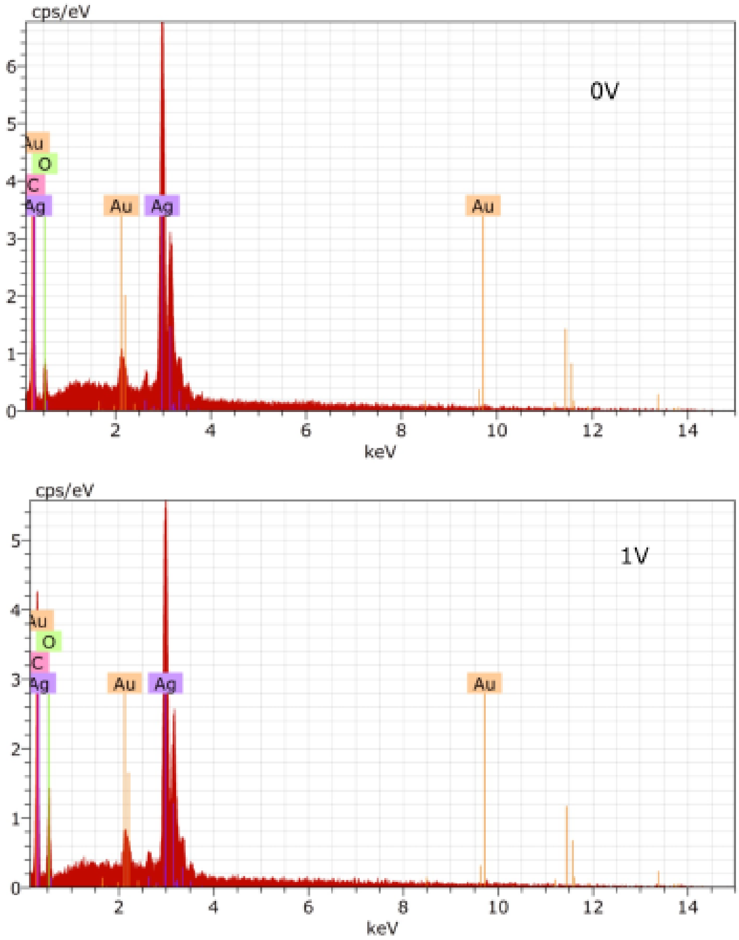

Figure S2. Energy-dispersive X-Ray spectra of the pristine electrode (0 V, top) and after application of 1 V (bottom).

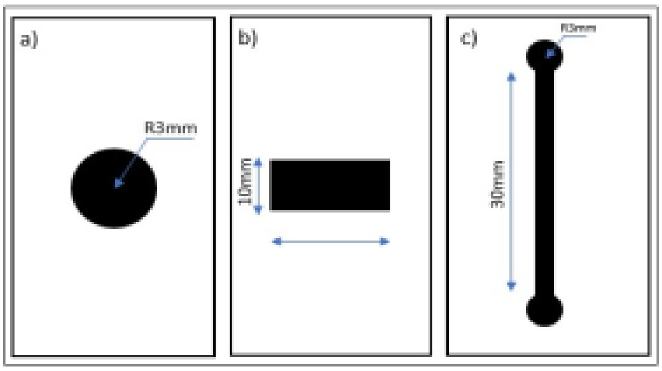

Figure S3. Electrodes configuration used for the experiments with a) and b) used for dimension characterization and c) for electrical characterization.

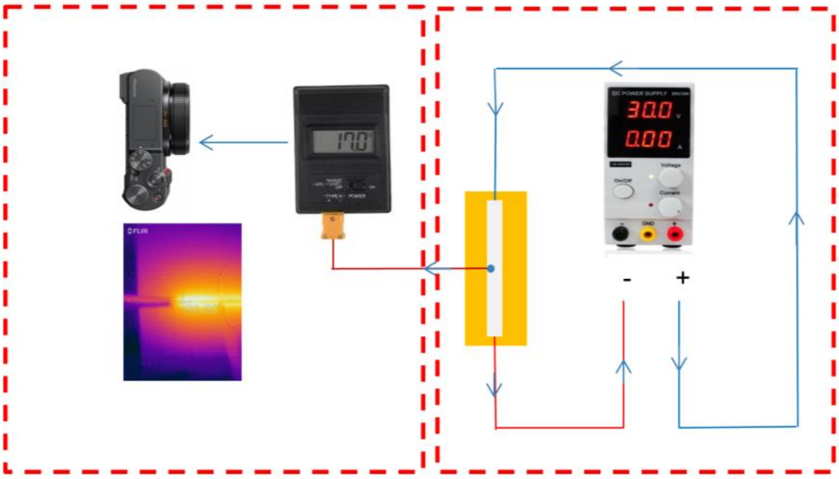

Figure S4. Temperature measurement set-up during heating.

Table S1. Correlation between applied current (constant current) and applied voltage (constant voltage) for 30% PVA nanocomposite. Measurements taken on 3 independent samples.

Current	Voltage (avg)	Voltage (SD)
0.2	0.073	0.02
0.4	0.123	0.03
0.6	0.2	0.04
0.8	0.29	0.02
1	0.38	0.03
2	0.473	0.07
3	0.513	0.02
4	0.607	0.01
5	0.812	0.01
6	0.940	0.07
7	1.093	0.10

During the experiments, some measurements were made by setting a voltage value (instead of constant current). For the sake of clarity and to improve readability, we have only reported current values in the manuscript since they were consistent with the set voltage values as shown in Table SM1. The measurements were done in triplicate on different samples and indicate a low standard deviation. Measurements affected include those reported in Figures 5, 7 and 8.
